# The Week's Work

**Published:** 1909-10-23

**Authors:** 


### The Week's Work.

THE Asylum Workers' Superannuation Bill, which was read a second time in the Lords recently, obtained valuable support in the Higher Chamber. It was expected by those in charge of the measure that it would receive some opposition from the county councils, but no fewer than nineteen of these bodies have already expressed themselves in favour of it. Objections have, it is true, been raised by a few councils, whose main contention is that the Bill, if it becomes law, will lay a still greater burden on the ratepayers than they have already to bear. That objection can be alleged against every salutary measure that has for its object the amelioration of anything and any condition in which the public as a whole ought to take an interest, and we do not think that it will have much force when the Bill comes up for final discussion in the House of Lords. There can be no doubt that it is a good Bill. It attempts to help asylum servants, whose lot hitherto has not been of the best, to make provision for themselves in their old age; and it is a measure, therefore, that should have the cordial support of everyone who is interested in, and acquainted with, the condition of asylum workers. In the debate on the second reading Lord Belper, speaking as Chairman of the County Councils Association, objected to the Bill on the ground that it was a compulsory measure. The whole subject of superannuation grants had been barely discussed in the Commons, where the Bill had been passed without opposition, and Lord Belper thought that it would be a mistake to accept the compulsory clauses without a full discussion on their merits. While not directly opposing the second reading, Lord Belper reserved his freedom of action to introduce amendments and to alter the measure before its final passage. Any tampering with the clauses of the Bill as they stand at present is to be deprecated. There is no time to discuss the whole question of superannuation grants with those who do not admit that such grants are just and reasonable; and Parliament has too much work before it this session to waste its time in debating theoretical improvements which will probably be thrown out in the Commons, even if accepted in the Higher Chamber. Everyone who has read the Bill must admit that it is a measure that can be cordially supported on its merits. It raises no new issues, and is not ultra-radical in its provisions. What it provides for is simply the improvement of the present unjust conditions under which asylum officers live and work.

THE aim of the measure is to provide assured pensions to officers and servants engaged in asylum work. It provides that such pensions should be raised on a contributory basis, and according to definite scales as laid down in the schedules. As a matter of fact, the measure has many points of similarity with the Poor Law Officers' Superannuation Act, and reminds one of the clauses introduced by Lord Halsbury into his Lunacy Bill ten years ago. The Bill has received the support of the various societies and associations that are competent to express an opinion on its merits, and has been before a select committee of the Commons, where it was fully discussed and read a third time. It is to be hoped that it will have a smooth and easy passage through the Lords and that there will be no delay in giving effect to its provisions.

IN the interview with Sir William Church, which appeared in our issue of last week, reference was made to the Provident Dispensary Association. This association, the full title of which is the Metropolitan Provident Medical Association, was founded almost 30 years ago, and was incorporated under the Companies Act in 1892. It has its offices at 5 Lamb's Conduit Street, Bloomsbury, and issues an annual report. Its objects are to provide upon principles of mutual assurance (by means of small periodical payments) efficient medical treatment and medicine for those members of the working classes and their families who are unable to pay the ordinary medical fees, and to co-operate with the governing bodies of the metropolitan hospitals in order that they may be relieved of the large number of ordinary cases of illness at present overcrowding the out-patient departments, and have referred to them from the provident dispensaries such cases as may need hospital treatment.

THESE objects are attained by the formation, in suitable districts, of provident dispensaries, as branches of the association. The dispensaries are under local management, to which the association cordially invites local practitioners to attach themselves. At present there are 20 such branches, 16 of which are dispensaries, while four are medical clubs. They have a membership of nearly 13,000 family and individual tickets. During 1908 the receipts from members were over £5,000. Of this amount the medical officers attached to the dispensaries received nearly £3,400, while fully 90 per cent. of the whole income was devoted to the medical part of the work. The association, it is gratifying to notice, is increasing in popularity. It works quietly, and is comparatively unknown to the mass of the public—a fact that may account for the small amount it annually receives from outside friends.

THE article in our last issue on the responsibility of silence, has attracted much attention. We have received several letters on the subject, which we hope to publish in a future number, as exigencies of space forbid their full publication this week. It is felt pretty generally by the best men and women in hospital work that what was urged must have weight and bear fruit. We dealt in that article with the responsibility of silence on the part of the resident and honorary medical staff. There is an equal responsibility on the part of the chairman and members of each hospital committee, though it is one which is seldom realised and still less often acted up to. We give two examples of the responsibility of silence on the part of a committee both in word and action, which will be seen to be onerous indeed. We shall be glad to have other instances and to hear the views of the chairman and of members of the committee upon this matter.

With the best intentions a hospital committee has sanctioned a considerable expenditure in connection with the new wing for a new hospital by agreeing to the introduction of the Plenum system of ventilation. That system, fortunately, and deservedly, is unpopular in this country, but it has found its way into one or two institutions, not to the advantage of the patients and to the discomfort of the staff. On Sunday we were in a large children's ward at Huddersfield Infirmary, a hospital which is beautifully kept, where the wards, it is said, are ventilated on the Plenum system. We spent some time in the children's ward studying the condition of the children and of the atmosphere. We came to the conclusion that every child in the ward was air-starved, and that it was a cruelty to treat children under conditions like those which prevailed in this ward. The same thing happened in similar conditions at St. Thomas's Hospital, London, and prompt steps were taken to have the windows opened and so to restore to the children the air of heaven which is the birthright of every little child. We hope that before the week is out the children's ward at the infirmary mentioned may be flushed with fresh air, and that fresh air may continually flow through it from that moment so long as it may be occupied as a children's ward. The responsibility of the committee in regard to this matter is onerous indeed. They have spent their money on an imperfect system, it is true, and will have to spend more to put matters right. But what is money when the welfare and even the life of little children may be at stake?

The second instance is even more remarkable, for it illustrates a want of apprehension and sense of proportion on the part of the particular committees which is extraordinary. A committee of an important hospital—which shall be nameless—had amongst its members a gentleman who was very wealthy and who gave large sums to the institution. Each member of the committee had a latch-key which enabled him to enter the hospital at all hours. The wealthy member was a dipsomaniac, and he used sometimes to come to the hospital in the night in search of alcohol. One night he stole into the Night Superintendent's room, shut the door, put his back against it, and demanded brandy. The night sister found herself cornered and was much frightened, when her visitor, a powerful man, took her by the shoulders and shook her severely, demanding alcohol all the time! Ultimately, by presence of mind, the night sister managed to get into the corridor and to close the door on her visitor, locking it on the outside. She then apprised the matron, who locked her apartments, a precaution which proved to be wisdom, for on being released, the night visitor rushed to the matron's apartments and endeavoured to force the door. He had ultimately to be removed from the hospital by force. On account of his wealth, the committeeman was not deposed, but the matter was overlooked. So the night staff were liable to the same kind of attacks for a considerable period afterwards, for the man in question had the habit of using his latchkey whenever he was at his worst. Here is an instance where the committee by silence committed a grievous moral wrong. True, the hospital ultimately received a large sum of money at the death of the dipsomaniac, but that constitutes no justification for the wrong action of this committee, each member of which failed to recognise the responsibility of silence. Every member of the staff of a great hospital should be supported and rendered secure from risks of this kind at whatever cost, real or imaginary.

A measure of some interest to hospital workers, the Health Resorts and Watering Places (Ireland) Bill, engaged the attention of the House of Lords on Tuesday last. The object of the Bill is to enable local authorities in Ireland to spend a small amount of money in advertising the advantages, from a health point of view, of their several localities. The presence of hospital accommodation of an up-to-date character is an important consideration with an invalid in deciding upon a health resort, and doubtless the Bill, if it becomes law, will lead to the improvement of cottage hospitals and convalescent homes in Ireland. At present there are health resorts in the best parts of Ireland where it is impossible for visitors, in urgent cases, to obtain hospital attendance or accommodation. However artistic and however excellent a country cottage may be, it is scarcely the place in which to conduct a major surgical operation. Local authorities on the Continent have grasped the importance of the fact that every health resort should have sufficient and efficient hospital accommodation, and, in Germany at least, it is now possible to secure such accommodation at even the smaller resorts.

At a meeting of the City Board of Guardians last week the question of hospital contributions was discussed on a motion by Mr. Lile to the effect that an annual subscription of twenty guineas should be made to St. Bartholomew's Hospital. In supporting the motion, it was pointed out that at the last meeting it had been proposed that various sums should be subscribed to charitable institutions in London, but that, for some reason or other, the name of St. Bartholomew's Hospital had not been included in the list. Thus it had been agreed to support the London Hospital, and it was therefore only fair that the large city hospital should not be forgotten. Some of the members, in the course of the discussion, denied that the Board derived any benefit whatever from the city hospital, although some benefit was undoubtedly derived from the East End institution. It was pointed out that many patients were sent from St. Bartholomew's to the infirmary and so were made a charge upon the city. In support of the motion, it was stated that both St. Bartholomew's and the Royal General Dispensary, which is practically a part of its outpatient department, relieved many poor patients who would otherwise become inmates of the infirmaries and so a burden on the rates. After an animated discussion the motion was put to the vote and lost, 10 members voting for it and 11 against.

